# Sex-based considerations for implementation of ventricular assist device therapy

**DOI:** 10.3389/fcvm.2022.1011192

**Published:** 2022-10-18

**Authors:** K. Candis Jones-Ungerleider, Angela Rose, Kevin Knott, Sarah Comstock, Jonathan W. Haft, Francis D. Pagani, Paul C. Tang

**Affiliations:** ^1^Department of Cardiac Surgery, School of Medicine, University of Michigan, Ann Arbor, MI, United States; ^2^Department of Cardiac Surgery, University of Michigan, Ann Arbor, MI, United States

**Keywords:** LVAD, sex, female, heart failure, right heart failure (RHF)

## Abstract

Women with advanced heart failure receive advanced surgical therapies such as durable left ventricular assist device (LVAD) implantation or heart transplantation at a rate much lower compared to males. Reasons for this discrepancy remain largely unknown. Much of what is understood reflects outcomes of those patients who ultimately receive device implant or heart transplantation. Females have been shown to have a higher mortality following LVAD implantation and experience higher rates of bleeding and clotting phenomena and right ventricular failure. Beyond outcomes, the literature is limited in the identification of pre-operative factors that drive lower than expected LVAD implant rates in this population. More focused research is needed to define the disparities in advance heart failure therapy delivery in women and other underserved populations.

## Introduction

Women represent ~30–50% of patients with stage D heart failure (1, 2), but account for only 21% of the patients receiving durable left ventricular assist device (LVAD) implants (3). Approximately 3,000 durable LVAD implants occur annually and the relatively low proportion of female recipients has remained relatively constant across eras (2010–2014 vs. 2015–2019). This disparity in women receiving durable LVAD therapy can be explained by a myriad of factors such as sex-based differences in disease biology, patient size considerations, comorbidities, pre-implant considerations and post-implant outcomes. Perhaps more difficult to delineate objectively are the less explicit contributions of system and provider bias that may result in fewer women ultimately receiving a device.

## Etiology of heart failure

The epidemiology of heart failure in women varies significantly from that of men and, therefore, disparities in the treatment of advanced heart failure are frequently attributed to distinct biologies. While the lifetime risk of heart failure is similar in males and females (4), heart failure with preserved ejection fraction disproportionally affects women and heart failure with reduced ejection fraction disproportionately affects men (5). Females account for ~30–40% of all systolic heart failure (6), with non-ischemic disease being the most common etiology. In contrast, ischemic cardiomyopathy is much more prevalent in males. Additionally, females are uniquely susceptible to other forms of heart failure such as peri-partem cardiomyopathy, chemotherapy (e.g., breast cancer therapy) induced cardiomyopathies, takotsubo cardiomyopathy, and autoimmune mediated disease.

## Demographic and comorbidity profile

There have been two large analyses of Interagency Registry for Mechanically Assisted Circulatory Support (INTERMACS) data that specifically address sex differences in utilization of LVAD therapy (7, 8). Both these studies report specific trends in the continuous flow device era where women who receive LVADs tend to be younger than their male counterparts. While the age of females receiving LVADs has been relatively stable across eras, men receiving devices in the continuous flow era tend to be older relative to the previous era of pulsatile devices. Females receiving durable LVADs are more frequently African Americans when compared to the male population. Further, female LVAD recipients are more likely to suffer from obesity, thyroid disorders, rheumatoid arthritis, collagen vascular disease, chronic blood loss anemia and depression, whereas men are more likely to experience diabetes, hypertension, coronary artery disease, prior coronary artery bypass grafting, chronic renal failure, and greater alcohol use. The primary payer is more likely Medicaid for females and the overall median household income is shifted toward the lower percentiles for female LVAD recipients in the continuous flow device era.

## Pre-implant considerations: From diagnosis to device delivery

Ventricular assist devices improve survival, functional status and quality of life. Although women in the greater heart failure community tend to have better survival, women report worse quality of life compared to age and ejection fraction matched male counterparts (9). While it is apparent that females have much to gain from advanced heart failure therapies, improving patient access to advanced surgical heart failure therapies is complex. Connecting female patients with advanced heart failure to advanced surgical heart failure therapies requires navigation of the greater healthcare system, medical decision making by many levels of providers, and the willingness of the patient to commit to the treatment plan.

There is a paucity of data to help assess potential sex differences in decision making at the clinician or system level. It is known that females are less likely to receive temporary mechanical circulatory support in the setting of cardiogenic shock (OR = 0.76); this is also true of black patients and those insured by Medicare and Medicaid (10). An additional challenge in understanding durable device delivery is the interplay of decision making with that of heart transplantation. There are unique barriers to heart transplantation in females including increased rates of obesity and allosensitization. In a study from Emory University assessing eligibility for advanced heart failure therapies, females were less likely to be eligible for heart transplant (21 vs. 47% of patients evaluated) and more likely to be recommended a VAD as destination therapy (24 vs. 9.7%) (11). Furthermore, a separate study demonstrated that females who underwent LVAD implantation were less likely to undergo subsequent transplantation and had higher transplant waitlist mortality (12).

Sex specific patient related treatment preferences may also contribute to differences in delivery of advanced heart failure therapies. Females have been shown to be less willing to undergo heart transplantation due to self refusal (13). Additionally, in a multi-institutional study of socioeconomic factors and patient preferences for LVADs, lower income and lesser education were associated with an increased willingness to undergo VAD implantation (14). While there was no discernable relationship between patient acceptance of a device and sex or marital status, only 25% of respondents were female.

## Outcomes with VAD

Outcomes following durable LVAD implantation have improved over time with trends following device innovation and market approvals. LVAD therapy outcomes are frequently stratified by device era, with distinction between the early pulsatile flow devices (pre 2008) and more modern continuous flow devices (post 2008) corresponding to regulatory approval of the Heartmate II for destination therapy. A further distinction of the continuous flow era can be made between continuous flow design including axial devices (i.e., Heartmate II, Abbott Labs, Chicago, IL) and centrifugal devices (i.e., Heartware HVAD, Medtronic Inc., Minneapolis, MN; Heartmate 3, Abbott Labs, Chicago, IL), with the regulatory approval of the Heartware HVAD for BTT in November 2012. This is particularly pertinent given a demonstrated era effect in improved survival when stratifying implants prior to and post 2013, which favors the more recent era (15). Lastly, in 2019 the MOMENTUM3 trial published 2 year data on the latest LVAD technology, the Heartmate III device, which was engineered for improved hemocompatibility. Use of these new, innovative pumps was associated with superior outcomes relative to predecessor devices, including most notably a dramatic reduction in rate of stroke (16).

Historically, broad utilization of pulsatile devices was often precluded in females due to smaller body habitus, as pulsatile LVADs were bulky and not suitable for patients with body surface areas < 1.5 m^2^. With FDA approval of the Heartmate II, an era of smaller and lighter continuous flow devices (17) became newly available to populations—in large part females—who were underserved by prior generations of mechanical support.

## Mortality

Sex related disparities in outcomes following VAD implantation are most pronounced in the pulsatile flow era, with more modern data of continuous flow devices being more nuanced. In an analysis of National Inpatient Sample data reflecting over 6,000 patients undergoing implants from 2004 to 2016, inpatient mortality in women during the pulsatile flow era was higher than that of men (47 vs. 31%); however, there was no discernable difference in the continuous flow era (13 and 12%, respectively) (18). An INTERMACS study reflecting nearly 2,000 patients, 400 females, spanning 2006 to 2010 showed no difference in mortality between females and males (16 and 17%) at average of 7 months of follow-up, irrespective of device type (8). In contrast, the eighth annual INTERMACS report published in 2017 suggests that among patients with continuous flow devices there is a higher risk of early (3 month) mortality in women (HR 1.47), potentially attributable to right ventricular failure and major bleeding events (15). Similarly, in a study of International Society for Heart and Lung Transplantation (ISHLT) Mechanically Assisted Circulatory Support (IMACS) data limited to the centrifugal era (post 2013), women had an increased risk of mortality (HR 1.36) attributable to an early hazard of death (HR 1.74 in the first 4 months) (19). Nearly a quarter of this increased mortality risk was mediated by pre-operative echo findings of smaller left ventricular volumes and increased tricuspid regurgitation (both surrogates for patient size and right ventricular function). Most recently, sub-analyses of Heartmate III clinical trial data suggests no difference in morbidity and mortality profile for this new generation device with no apparent difference in the composite outcome of survival out to 2 years free of stroke or reoperation for malfunctioning device across sex (16).

## Morbidity

There is a differential morbidity profile following VAD implantation in females largely attributable to bleeding, thromboembolic phenomena and right ventricular failure. The origins of dysregulated coagulation in females have been explored through a broad range of investigations, including estrogen effects on clotting factors, sex differences in pharmacokinetics and dynamics of anticoagulants, as well as lower pump speeds, particularly in the era of the Heartmate II.

### Neurological events (stroke)

Females who undergo LVAD implantation are at higher risk of neurologic events than their male counterparts. Early data with the Heartmate II highlighted an increased risk of hemorrhagic, but not ischemic stroke in females (20) (incidence 12 vs. 3%, 0.1 vs. 0.04 events per patient year). This risk of increased hemorrhagic stroke in women remained a trend even after matching for body surface area (BSA). In an analysis of 900 Heartmate II recipients (23% female) and risk factors for stroke, female sex was associated with a hazard ratio of 1.92 for hemorrhagic and 1.84 for ischemic stroke (21). More recent INTERMACS data support that women may have an intrinsically increased risk of stroke, even after adjusting for common risk factors. Females were found to have a shorter time to first neurologic event in the pulsatile flow era and a trend toward the same in the continuous flow era. This difference occurred even while the overall rate of neurologic events declined significantly with continuous flow devices (8). Heartmate III data suggests an overall decrease in stroke risk with this contemporary device; outcomes by sex however are limited to the primary composite end point, which incorporates neurologic morbidity, for which there was no discernable difference.

### Bleeding

A number of studies indicate that women are at higher risk of bleeding events both in the immediate post-operative period and more chronically while on anticoagulation therapy. A review of European Registry for Patients with Mechanical Circulatory Support (EuroMACS) data of 966 patients receiving a durable VAD (151 women) revealed that females were twice as likely as males to have a major bleeding event as defined by INTERMACs adverse event definitions. This applied for the first 30 days of post-implant period and also identified twice as many bleeding events per patient year compared to males (22). A single center study of 375 recipients (84 females) receiving continuous flow devices observed that, although mediastinal bleeding was similar between men and women in the immediate post-operative period, women had a 60% higher hazard of overall bleeding complications. This was largely driven by clinically significant mucosal (gynecologic and oronasal pharyngeal) bleeding occurring after the first 30 days post implant but prior to the 1st year (23). Ten percent of all females in this study experienced gynecological bleeding with a need for transfusion or surgical intervention. A separate study noted that freedom from gynecological bleeding was 84% at 1 year and 73% at 2 years for females on long term LVAD support. Gynecological-bleeding after LVAD implantation in this study was defined as needing emergency outpatient visits, hospitalization, blood transfusions, hormonal therapy, and/or surgery (24). In larger datasets, females do not appear to be at higher risk for gastrointestinal bleeding, although there are limited reports which suggest that gastrointestinal bleeding risk is lower in males (25).

### Right ventricular failure

Right ventricular failure is more common in women potentially due to a greater occurrence of non-ischemic cardiomyopathy, later presentation of disease, and higher incidence of arrhythmias (26). In a systematic review with meta-analysis of sex specific differences in outcomes for patients receiving continuous flow LVADs, females were 2.12 times more likely to develop right heart failure necessitating right ventricular assist implantation (27). Similarly, a study of EuroMACS data from 2011 to 2014 revealed that right ventricular failure, defined as requiring additional temporary right ventricular assist device (RVAD) support, occurred at a rate of 0.11 events per patient year in females and 0.04 events per patient year in males (22). Women who underwent RVAD implantation had a higher probability of death compared to isolated LVAD, but mortality following RVAD implantation was not significantly different across sex distributions.

## Discussion

Women reflect a minority proportion of patients receiving advanced surgical heart failure therapies. There is a discrepancy between the high burden of heart failure in females and the number of females who ultimately receive LVAD therapy. Females who present for advanced heart failure therapies have a disparate preoperative profile compared to male counterparts, including the very nature of the underlying heart disease and associated comorbidities. Additionally, sex differences in pre-implant factors such as candidacy for transplantation and decision making at the patient, physician, and systems level may impact presentation for surgical evaluation and patient willingness to accept a device when offered. Much of what is known about LVADs in females with advanced heart failure is derived from the endpoint of device delivery, particularly at the clinical trial level; female sex has been associated with a historically higher rate of mortality and higher incidences of stroke, bleeding, and right ventricular failure following implant. While the representation of females within VAD study cohorts has increased with advances in technology, heart failure therapy and device trials overall continue to underrepresent females, with participation by females far lagging prevalence of the disease by 0.55:1.0 (28). Newer data reflecting outcomes with the Heartmate III is encouraging however sub-analysis by sex is limited and interpretation should be measured given females overall still represent a small proportion of the study cohort. Outcome measures across large datasets such as INTERMACS, EuroMACS, National Inpatient Sample (NIS), and single institutional study cohorts support sex related gaps demonstrating less favorable morbidity and mortality in females. Although sex-based data often were obtained from powerful databases with large populations, there is insufficient granularity to provide specific insight into the likely multifactorial reasons for these observations. Smaller, more comprehensive datasets are limited by power, regional differences in patient populations, and institutional practices and physician preferences that may not be universal. In the absence of datasets specifically built to address disparities, it is often inferred that the origins of sex related discrepancies following LVAD implantation are attributable to differences in biology and physiology such as heart failure etiology and comorbidity profile.

**Figure 1:**
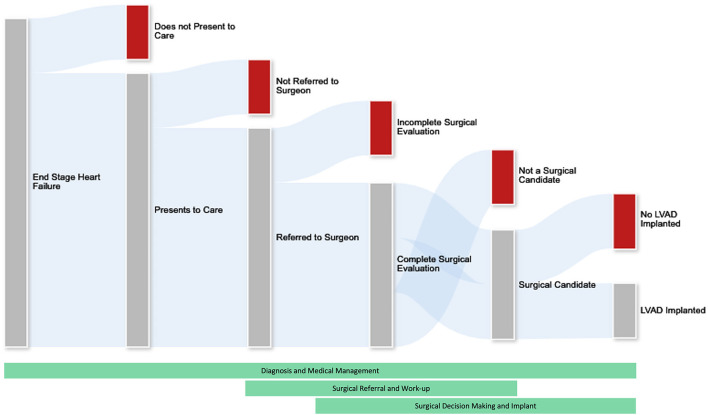
The process of patient attrition in the ability to deliver advanced heart failure therapies. Not all patients with end stage heart failure will undergo advanced heart failure therapies. We must understand the processes of diagnosis, medical management, referral for surgery, and surgical decision making to better address disparities in care. This is challenging as attrition of would-be surgical candidates occurs throughout the healthcare system and across medical and surgical specialties. Future research efforts should focus on medical and socioeconomic aspects of this attrition including but certainly not limited to physician bias, system factors, and patient preferences and limitations.

The treatment algorithm connecting a woman in the community with heart failure with successful LVAD implantation is complex. Patients with heart failure should be appropriately identified, medically managed, referred for surgical evaluation and ultimately undergo treatment. There is a critical knowledge gap in understanding the true number of females who could be eligible for or benefit from advanced heart failure therapies out in the community. Our current understanding of advanced heart failure care in females mainly reflects the outcomes of those patients who were able to obtain mechanical support or heart transplantation. To address disparities in access to care, we should understand the processes of advanced heart failure diagnosis, medical management, referral for surgery, and surgical decision making, as well as how each of these processes may be uniquely influenced by sex (Figure 1). Developing appropriately specific tools to collect data illuminating disparities across LVAD therapy delivery stands to benefit women and other underserved populations within the advanced heart failure community.

## Author contributions

KJ-U, AR, KK, SC, JH, FP, and PT contributed to conception and design of the review. KJ-U wrote the first draft of the manuscript. KJ-U, FP, and PT wrote sections of the manuscript. All authors contributed to manuscript revision, read, and approved the submitted version.

## Funding

This study was partially supported by the National Institutes of Health grant HL164416 awarded to PT.

## Conflict of interest

The authors declare that the research was conducted in the absence of any commercial or financial relationships that could be construed as a potential conflict of interest.

## Publisher's note

All claims expressed in this article are solely those of the authors and do not necessarily represent those of their affiliated organizations, or those of the publisher, the editors and the reviewers. Any product that may be evaluated in this article, or claim that may be made by its manufacturer, is not guaranteed or endorsed by the publisher.
